# A history of liaison psychiatry in the UK

**DOI:** 10.1192/pb.bp.116.053728

**Published:** 2016-08

**Authors:** Peter Aitken, Geoffrey Lloyd, Richard Mayou, Christopher Bass, Michael Sharpe

## Abstract

**Aims and method** To record the development of liaison psychiatry in the UK and to summarise the current levels of activity. We also highlight the challenges the specialty may face if it is to develop further. History since the 1970s is reviewed by early pioneers and those involved in the present day, with a focus on the key role played by members of the Royal College of Psychiatrists.

**Results** We describe the development of training guidelines, the publication of joint documents with other Royal Colleges, establishing international collaborations and defining service specifications. We emphasise the importance of collaboration with other medical organisations, and describe successes and pitfalls.

**Clinical implications** Much has been achieved but challenges remain. Liaison psychiatry has a potentially important role in improving patient care. It needs to adapt to the requirements of the current National Health Service, marshal evidence for cost-effectiveness and persuade healthcare commissioners to fund services that are appropriate for the psychological needs of general hospital patients.

The psychological and psychiatric care of people with physical complaints has always been an essential part of good medical practice and, in the early 20th century, became the focus of a small medical subspecialty called psychological medicine. This article records the evolution of the part of psychiatry that serves people with medical illnesses and looks to its future, as medical services, training and expectations of care evolve in the UK.

## Liaison psychiatry in the UK

Until the 1970s dedicated liaison psychiatry services were virtually unknown in the UK, although they had already become widely established elsewhere, particularly in the USA.^1^ Indeed, liaison psychiatry, the term allegedly introduced to describe the department at Colorado General Hospital in Denver,^2^ was in its early years predominantly an American movement,^3^ much influenced by now-discredited psychosomatic theories of illness.^4^ It flourished in America in the 1970s in part as a result of federal funding to support its role in medical student education. Most American services provided in-patient consultation to patients but there were also those who believed in the importance of liaison with medical teams. The sometimes heated theoretical arguments about the relative merits of these activities led to the adoption of the cumbersome, combined term consultation-liaison (C-L) psychiatry.

Awareness of these American developments and the establishment of new psychiatric units within general hospitals meant that more psychiatrists became interested in liaison psychiatry in the UK and a small number of designated posts were created. However, the crucial event enabling the development of a recognised subspecialty of liaison psychiatry was the foundation of the Royal College of Psychiatrists in 1971, bringing together the asylum doctors (‘alienists’) and the teaching hospital psychiatrists. Following the examples of the older Royal Colleges, the Royal College of Psychiatrists formed specialist sections and took on training and advisory planning roles. But the old teaching hospital ethos of psychiatry as a part of medicine was progressively lost and the new College did not initially cater for clinicians who wanted to specialise in psychiatry of people with medical illnessses.

## The Royal College of Psychiatrists' Liaison Psychiatry Special Interest Group

However, all was to change. Informal discussions were begun among a small group of interested clinicians, with Richard Mayou and Geoffrey Lloyd as prominent members. These resulted in an agreement to explore the possibility of establishing a group for those with interest in medical illnesses within the College. The response to a preliminary letter in the *Psychiatric Bulletin*^5^ revealed considerable enthusiasm for a forum to facilitate discussion of clinical, research and teaching interests. Sir Desmond Pond, College President from 1979 to 1982, encouraged these aspirations. A preliminary planning meeting was held during the College Quarterly Meeting in Oxford in 1983, followed by a session at the Annual General Meeting in Edinburgh in 1984. An application was made to the College to establish a special interest group and this was approved, although not without opposition from some senior fellows who did not regard liaison psychiatry as a special field of activity separate from general psychiatry.^6^ Although the College Council did not realise it, this initial and modest special interest group was always seen by the protagonists as the first step towards liaison psychiatry becoming a fully-fledged subspecialty.

The name of the new special interest group provoked some controversy. Psychosomatic medicine was considered to have too strong a link with past, unproven aetiological hypotheses whereas psychological medicine, the preferred term, had recently been taken to name a new general psychiatry journal. Although the name ‘liaison psychiatry’ was not considered ideal, it had the advantage of familiarity and had already gained acceptance in the USA. The self-appointed steering group became the first Royal College of Psychiatrists' Liaison Psychiatry Committee. Richard Mayou, who had led much of the preliminary work, was elected chairman, holding this post until 1989. Looking back, the central importance of a succession of enthusiastic activists in establishing the subspecialty is very clear. All these activists were relatively young, ambitious for their subject and not overwhelmed by College bureaucracy. We had two related aims: first, to create a psychiatric subspecialty that required specialised expertise and training, and second, to promote psychological understanding and care within all general hospital medical practice. These aims were to be achieved in a variety of ways (Box 1).

**Box 1** Setting up the subspecialty of liaison psychiatry – preliminary tasks

- Holding meetings and conferences to get people together
- Conducting surveys to find out what was happening
- Writing guidelines for training
- Doing research to provide an evidence base
- Teaching others
- Making international links to share knowledge
- Establishing collaborations with other medical organisations
- Setting out service specifications

## Setting up the specialty: tasks of the liaison psychiatry special interest group

### Meetings and conferences

From the very beginning, the special interest group organised sessions both at College meetings and its own conferences. Its first residential conference, held in Oxford in 1987, was a great success; a second successful Oxford conference in 1989 included European and American colleagues. Subsequently, the liaison psychiatry conference has become an established regular feature of the College calendar and attracts a growing audience from members within the UK and abroad. Its success has led to joint meetings with colleagues in The Netherlands, Denmark, Spain and Portugal.

### Surveys

Once the group had been recognised it proved surprisingly easy to achieve a substantial membership. A survey of members found that there was in fact more clinical and academic activity within liaison psychiatry than had been realised, but also that there were few specific posts. Respondents to these surveys frequently complained there was insufficient time to carry out their work satisfactorily.^7^ They also showed that services had developed unevenly and that few health districts had given priority to developing liaison psychiatry. Subsequent surveys documented slow progress, for example Guthrie,^8^ until a recent expansion of posts probably influenced by the cost-effective analysis of the RAID service in Birmingham (discussed in Successes).

### Training guidelines and training posts

When the Royal College of Psychiatrists was founded in 1971 there was no accredited higher training in psychiatry. The liaison psychiatry group wanted to emulate the College sections that had set out recommendations for accreditation, as this would confirm its separate expertise. After much consultation, and some battling against significant opposition, the group eventually persuaded the College Council to accept modest proposals for training.

The establishment of training posts in liaison psychiatry was the next priority. Requirements for training were agreed, published^9^ and later consolidated in a College publication,^10^ which also advised on setting up services and on teaching and research opportunities. The document included contributions from child and old age liaison psychiatrists. It was recommended that every psychiatry training scheme should have at least one post in liaison psychiatry at senior house officer or registrar level and that the trainee should have regular supervision and also access to systematic teaching. Trainee posts at senior registrar (specialist registrar) level were also to include experience of research, audit and teaching junior hospital doctors and medical students.

From 1992 Francis Creed and Elspeth Guthrie, both active early members of the special interest group, have run an annual advanced residential course held every June in Manchester. This attracts up to 36 trainees from the UK and abroad and feedback suggests that it has had an important role in career development.

### Research

Liaison psychiatry has always had a strong link with academic psychiatry, particularly in its early days. Indeed, many of the early advocates of the activity were academics. There was active and productive research activity in the universities of London, Manchester, Oxford, Edinburgh and Leeds. Areas where British research achieved international recognition included the study of suicide and self-harm, chest pain, depression in relation to stroke and cancer, and in communication skills training. Indeed, many of the earlier exponents of liaison psychiatry held academic posts. The past 20 years have, however, seen some diminution in the number of academics active in research directly relevant to liaison psychiatry. This may in part be because of the divergence between clinical provision and research in medicine in general and in part because psychiatric research has an increasing focus on biology and brain imaging. Nonetheless, the UK continues to have an internationally recognised research output relevant to liaison psychiatry and at least 10 professors of psychiatry who would see liaison psychiatry as their clinical specialty.

### Teaching

Historically, the teaching of medical students was a major part of the work of a specialist in psychological medicine in the London teaching hospitals. While liaison psychiatry offers huge potential to teach medical students about psychiatry in the context of general medical practice, rather than as a separate and arguably less generalisable activity, it has been slow to develop a commensurate role. However, the current crisis of recruitment into psychiatry and the relative accessibility of liaison psychiatry to medical students is encouraging an increase in the use of liaison psychiatry placements for medical students and, indeed, postgraduate training. There is probably still unfulfilled potential.

### International links

From the late 1970s onwards many individuals had contact with our colleagues in the USA. A number of UK academic liaison psychiatrists (including Mayou, Creed and Sharpe) established close personal relationships with and received warm acceptance from colleagues in the American Academy of Psychosomatic Medicine. Although the American healthcare system is in many ways substantially different from that in the UK, the contrast between the National Health Service (NHS) and the American system is potentially a creative one for thinking about how we can best deliver psychiatry into general medical settings. The link with the Academy remains strong and fertile and could be developed further.

There have also been productive links with Europe. In 1987 Frits Huyse, a Dutch liaison psychiatrist, invited Mayou and Lloyd to represent the College at the inaugural meeting of a European group for which he had obtained funding. This meeting led to enduring professional relationships, joint meetings and collaborations which enriched our activities. The Dutch experience was particularly influential in obtaining national funding to set out the specifications of an effective liaison service in every hospital. These informal meetings also led to seven UK liaison psychiatry services being included in the large European Consultation-Liaison Workgroup (ECLW) Collaborative Study.^11^ This involvement meant that the UK was well represented in the subsequent European Association for Consultation-Liaison Psychiatry and Psychosomatics (EACLPP), which later merged with the European Conference for Psychosomatic Research to form the European Association of Psychosomatic Medicine (EAPM).

### Collaboration with other medical organisations

From the beginning priority was given to working closely with other medical organisations as well as with other areas of psychiatry; the top priority was the Royal College of Physicians. Richard Mayou met with their president Raymond Hoffenberg in 1980 and established collaboration, resulting in joint conferences on medical symptoms not explained by organic disease^12^ and on psychiatric aspects of physical disease.^13^ The report of a joint working party on psychological care in general medical practice, mainly written by Francis Creed,^14^ can now be seen as a landmark in the development of the specialty. A subsequent joint conference was held in 2003 and a report of a joint working party chaired by Geoffrey Lloyd was published.^15^ The aims of these joint reports were to identify those areas of medical practice where psychiatry made a particular contribution to patient care, to review the effectiveness of psychological and psychopharmacological treatments in medical patients, and to encourage the establishment of effective general hospital liaison services.

Other reports have involved collaborative working parties with other organisations. These include the Royal College of Surgeons,^16^ chaired by Christopher Bass, the British Association of Accident and Emergency Medicine,^17^ chaired by Paul Gill, and the Royal College of General Practitioners,^18^ chaired by Else Guthrie. Another collaborative venture, in which the current College President Simon Wessely took a lead role, led to the publication of a joint report on chronic fatigue syndrome in 1996.^19^

Other initiatives include a collaboration of neuropsychiatrists, neurologists and psychologists to develop methods of assessing and treating patients with functional neurological symptoms – the UK Functional Neurological Symptoms (UK-FNS) group.^20^

### Service specifications

These joint reports set out for the first time recommended staffing requirements for a district hospital liaison psychiatry service. At the time the reports were written very few hospitals met the minimum recommended standards. Since the mid-1990s there has been an evolving consensus among clinicians that a liaison psychiatry service should be based within the hospital it serves and be managed and governed as part of that hospital trust. It has become clear that the level of staffing must depend on the size of hospital and the complexity of clinical problems it presents but also that a minimum service should include a whole-time consultant liaison psychiatrist, supported by two nurses who are capable of working autonomously, and a clinical psychologist who can provide supervision, training and delivery of brief psychological treatments, irrespective of geographical catchment area issues. Increasingly, expertise in old age psychiatry is essential, given that the majority of general hospital in-patients are now over the state pensionable age. Anderson & Ooman^21^ have cited evidence to show that, in this age group, working closely and collaboratively provides greater benefits than simple consultation on a case-by-case basis. At the other end of the age spectrum it has become clear that a specialised child psychiatry service is required in hospitals with paediatric departments.^22^ Putting these initiatives together we move toward the provision of liaison psychiatry ‘across the lifespan’.

## Successes

There have been many successes since the 1970s. These include the recognition of the specialty by the Royal College of Psychiatrists when it conferred section status in 1997 and faculty status in 2004 (faculty membership has since grown to over 4000). The passing years have also seen increasing attendance at faculty conferences and recently a rapid growth in the number of consultant posts. The clinical need for liaison psychiatry services has long been apparent and there is a growing body of evidence to support the clinical effectiveness of pharmacological^23^ and psychological treatments, particularly cognitive-behavioural therapy (CBT),^24^ in liaison psychiatry. Large-scale studies have demonstrated the effectiveness of integrated care in treating depression in patients with cancer^25^ and of CBT in improving symptoms of chronic fatigue.^26^ Schroder *et al*^27^ have shown the effectiveness of CBT in a range of functional somatic symptoms. One notable study demonstrated the cost-effectiveness of psychological treatment of functional bowel disorders,^28^ and has featured prominently in the National Institute for Health and Care Excellence (NICE) guidelines.

However, the evidence for the cost-effectiveness of a liaison service was unimpressive until Parsonage & Fossey^29^ conducted an economic evaluation of the Rapid Assessment Interface and Discharge (RAID) team established at the City Hospital, Birmingham by Tadros and colleagues. The evaluation concluded that the savings to the hospital in terms of reduced bed use (£3.55 million per year) were more than four times the cost of establishing the service (£0.8 million per year). Although it has methodological limitations, this study was important in supporting the recent development of liaison psychiatry services.

### The rise, fall and rise again of the Oxford service

Development since the 1970s has not always been linear, and the Oxford service provides an example. In the early 1970s liaison psychiatry began in Oxford as an innovative and pioneering service with a major focus on the emergency department and self-harm. Over the following decades it developed to provide a showcase, hospital-wide liaison psychiatry service linked to the academic department and training many doctors and nurses in the practice of psychiatry in the general medical environment. However, in the early years of the 21st century difficult relations between the mental health and acute trusts in Oxford led to the near demise of the service. Despite the apparent demolition, the foundations were sufficient to allow it to be reborn as a large, innovative, fully integrated psychological medicine service established as an integral part of the Oxford University Hospitals NHS Foundation Trust (the acute trust), again with strong academic links. This roller-coaster tale of a major teaching hospital has both reflected and influenced the morale of UK liaison psychiatry. Although it has a happy ending, the story to date is salutary in telling us that we cannot assume that the current enthusiasm and development in the area will be sustained indefinitely, particularly if we are unable to demonstrate its value through providing robust research evidence and routine clinical outcomes.

## Remaining challenges

Liaison psychiatry services in the UK remains patchy, with huge variations in levels of staffing between hospitals. Encouragingly, it is current government policy to establish a liaison psychiatry service in every general hospital.

A major obstacle to the development of such services remains in the almost entirely separate commissioning and delivery of mental health and physical healthcare. This separation institutionalises the unhelpful mind and body split.^30^ Indeed, developing services that offer parity for patients' mental and physical needs and deliver care in an integrated and seamless fashion is virtually impossible when there is such administrative separation.^31^ This reflects the problem that liaison psychiatry was created to solve and leaves it uncomfortably positioned between psychiatry and medicine, sometimes not fully accepted by either. However, the developing awareness that liaison services work most effectively when fully embedded in the acute hospital that they serve provides some optimism for addressing this challenge.

The need to enhance acute hospital patients' experience of medical care is undeniable and has been underlined by numerous reports of substandard care, most notably in the Mid Staffordshire NHS Trust.^32^ Liaison psychiatry has a potentially important role in improving care, but if it is to do so, several additional challenges will need to be addressed.^33^ In particular, effective collaborations will need to be established with other healthcare professionals, not only in nursing and psychology but also with those working in palliative care and pain services. Furthermore, given that acute hospital stays are now much shorter than they used to be and that more patients are being treated as day patients or out-patients, liaison psychiatry needs to adapt by extending into out-patient settings as well as in the community, alongside general practitioners.

The most recent College report on liaison psychiatry^34^ has recommended minimum levels of staffing that are unlikely to be met by many acute trusts in the near future. Consequently, liaison psychiatry's advocates need to continue to marshal evidence for both the clinical and cost-effectiveness of their work and to persuade healthcare commissioners to fund services that are appropriate for the psychological needs of general hospital patients.

Looking back, we can see that service development has always depended to a large extent on local clinical leaders. This is likely to continue. Morris^35^ and Butler & Temple^36^ have written helpful accounts of how they successfully overcame the challenges we have outlined here. Liaison psychiatry is poised to play a key role in addressing the need for more integrated care^37^ and in doing so increasingly re-adopting the original name of psychological medicine. We have come a long way from the early days of psychological medicine. And there remains much to do.
